# Supplementary material for: Structure and Rheological Properties of Glycerol Monolaurate-Induced Organogels: Influence of Hydrocolloids with Different Surface Charge
Source: Molecules. 2020 Nov 4;25(21):5117. doi: 10.3390/molecules25215117 (PMC7662997; doi:10.3390/molecules25215117)
Supplement: Supplementary file 1 [file molecules-25-05117-s001.pdf]

Figure S1 DSC curves of OG and MOGs

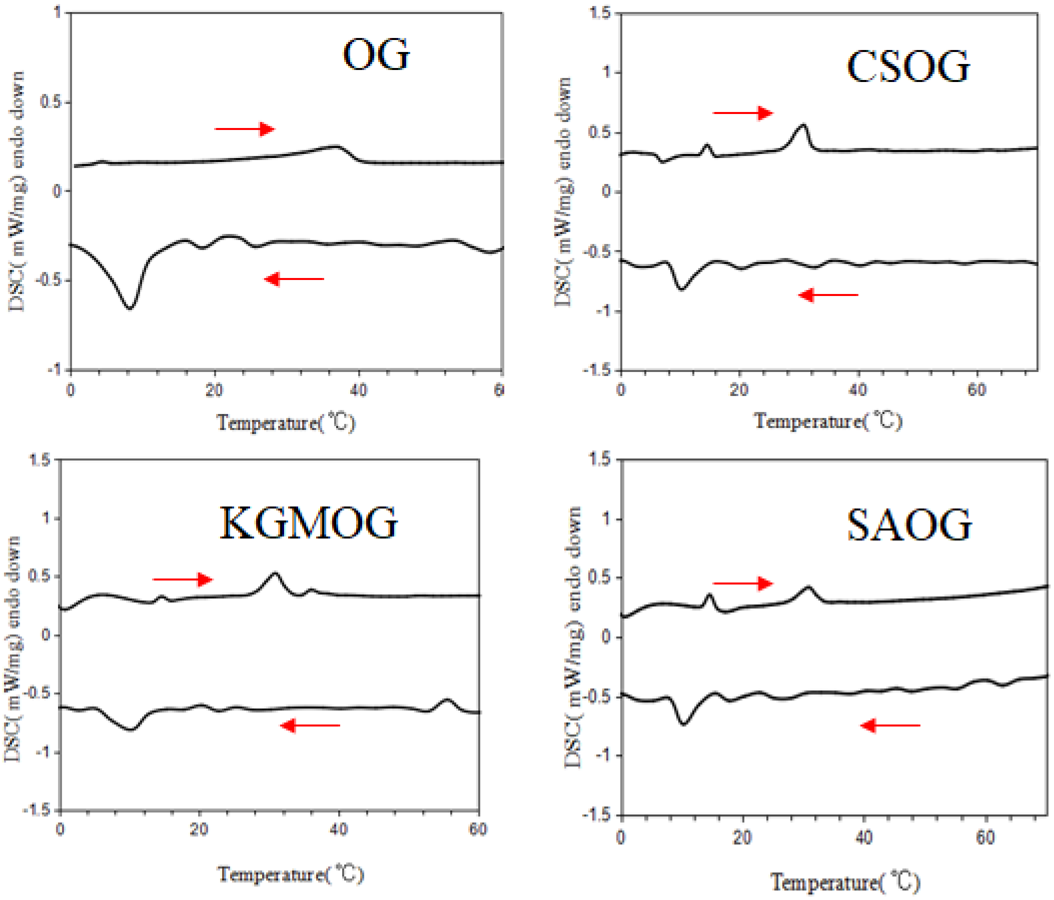

Table S1 Melting and crystallization temperature of OG and MOGs

Sample	$T_{onset, m}$(℃)	T_m	$T_{onset, c}$(℃)	T_c
OG	34.7	37.2	10.9	8.4
CSOG	27.9	31.9	15.3	10.1
KGMOG	27.2	30.9	13.2	10.2
SAOG	28.2	31.2	12.8	10.2
